# Malnutrition: An underlying health condition faced in sub Saharan Africa: Challenges and recommendations

**DOI:** 10.1016/j.amsu.2022.104769

**Published:** 2022-09-22

**Authors:** Adedoyin John-Joy Owolade, Ridwanullah Olamide Abdullateef, Ridwan Olamilekan Adesola, Esanju Daniel Olaloye

**Affiliations:** Faculty of Pharmacy, Obafemi Awolowo University, Ile Ife, Nigeria; Faculty of Clinical Sciences, College of Medicine, University of Ibadan, Ibadan, Oyo State, Nigeria; Department of Vertinary Medicine, Faculty of Vertinary Medicine, University of Ibadan, Nigeria; Faculty of Clinical Sciences, College of Medicine, University of Ibadan, Ibadan, Oyo State, Nigeria

**Keywords:** Malnutrition, Sub saharan Africa, Health

## Abstract

In Sub-Saharan Africa, the morbidity and mortality rate from malnutrition is increasing more than in any place in the world. Malnutrition has been a public health challenge in Sub-Saharan Africa that has not received enough attention. This commentary discussed the causes, effects and the need to prioritize the prevention and control of Malnutrition in Africa, together with practical recommendations. Several factors contribute to the high prevalence of malnutrition in sub-Saharan Africa. Some are poverty, overpopulation, unsuccessful small-scale agriculture, low educational status, climate change, corruption, wars and conflicts, fluctuation of food prices, etc.

Conversely, the effects of malnutrition on individuals have resulted in the development of illnesses and chronic health issues. Hence, there is a need to reach out to malnourished individuals, encourage the government, investors, and non-governmental organizations (NGOs) to take action, educate health staff to detect and react to early indicators of malnutrition, enhance agricultural product output, manage and preserve the environment, and use technology to its full potential. All of these suggestions will significantly impact the incidence of malnutrition in Sub-Saharan Africa.

## 1 Introduction

Malnutrition is a global problem with enormous social and economic implications and is the leading cause of illness worldwide. Malnutrition impacts physical and mental growth, immunity, and general health, limiting one's ability to achieve their full potential. Malnutrition affects one out of every three people, and almost every nation in Sub-Saharan Africa is dealing with a significant public health problem due to malnutrition [1]. The number of malnourished people in Sub-Saharan Africa has risen from 5.5 million to 30 million in the last decade, resulting in the death of over 3.5 million children under the age of five per year owing to insufficient food intake [2]. As of November 2021, Malnutrition has afflicted around 26%–38% of children under the age of five in selected African nations [3]. With the population likely to grow rapidly in the near future, this statistic has the potential to skyrocket.

Even though most countries in Sub-Saharan Africa have begun to progress toward the ultimate goal of reversing the trend of all kinds of Malnutrition by 2030, most efforts are not progressing at the appropriate rate [4]. All of this demonstrates that Malnutrition remains an underlying health condition in Sub-Saharan Africa, requiring more efforts to eradicate it.

## 2 Causes, impacts and consequences of malnutrition in sub Saharan Africa

Malnutrition has been identified as one of the major challenges facing Africa, especially the sub-Saharan region. The prevalence of undernutrition in the region was estimated to rise from 181 million in 2010 to about 222 million in 2016 [5]. In 2020, up to 264.2 million people living in sub-Saharan Africa were undernourished. That is about 24.1% of the population, the highest prevalence anywhere in the world [6].

Several factors contribute to the high prevalence of malnutrition in sub-Saharan Africa. Some are poverty, overpopulation, unsuccessful small-scale agriculture, low educational status, climate change, corruption, wars and conflicts, fluctuation of food prices, etc. [7].

Poverty is a significant factor contributing to the burden of malnutrition in sub-Saharan Africa. This is not surprising because the region is home to the highest impoverished people, with up to 413 million living on less than $1.90 a day [5]. 40–50% of the people in sub-Saharan Africa live below the poverty line [6]. Financial incapability implies that people have to feed on whatever they find while giving little or no regard to the nutritional composition. Because they have a lot of other financial obligations, they allocate very little money to acquire food. The consumption of nutrient-poor foods over time leads to malnutrition.

Overpopulation exists in several parts of sub-Saharan Africa, which is evident in the region's high number of births yearly. Many nuclear families are large in number, but the children depend on the meagre income their parents make. As a result, the few available resources are not enough to cater to the family's basic needs, including purchasing quality food. They, therefore, resort to the consumption of cheap foods, which often do not contain the essential nutrients. This ultimately leads to malnutrition. Illiteracy or low educational status, which is also prevalent in Africa, often implies that parents do not have adequate knowledge of the required nutrients they should provide for themselves and their wards. Even when resources are available to purchase good food, they might still not consume essential nutrients in the proper proportions. Corruption is another widespread problem in many countries in sub-Saharan Africa. When the government fails to ensure a stable economy, the lower and middle-class citizens suffer greatly. Many of them find it difficult to afford good food. A poor economy also leads to constant fluctuation in the prices of food items.

Malnutrition has led to the development of illnesses and chronic health issues due to its impacts. Every organ system in the body is affected by malnutrition in terms of function and recovery. The most visible indicator of malnutrition is weight loss owing to a loss of fat and muscle mass, including organ mass. Malnutrition also leads to a weakened immune system, impaired cognitive function and impaired development. Malnutrition has psychological and physical ramifications, such as lethargy, melancholy, anxiety, and self-neglect, and several consequences, some of which include an increased risk of obesity, heart disease, and diabetes [8].

## 3 Recommendations

In 2020, it was estimated that about 21% of the African population was suffering from malnutrition. That is about one in five people [9]. The number continues to increase every year. This implies that it is a great menace that requires adequate attention and prompt action by all stakeholders. These include individual citizens, healthcare workers, food producers and processors, government officials, etc.

### 3.1 Reaching out to those who are suffering from malnutrition

One way to tackle malnutrition is to devise means of reaching malnourished people, especially those in rural communities. Access to them can often only be gotten through physical means because most of them do not have access to the internet. This can be achieved by carrying out a series of community outreaches to their communities. The outreaches aim to carry out assessments, provide treatment, and advise on preventive measures. The team organizes occasional visits to the identified communities and provides care and support to affected individuals, especially children. The care and support can include food, nutritional supplements, medical treatment, psycho-social support, nutrition education, sanitation advice, etc. A project of this kind requires the collaboration of community volunteers, local health workers, and local health authorities.

### 3.2 Training health workers to detect malnutrition

Another approach to this problem is training community health workers and other health workers to recognize and respond to the early signs of malnutrition. This helps to reduce the number of severe cases of malnutrition that occur. Children who show early signs of malnutrition should be given supplementary food, vitamins, and supplements. Pregnant women at risk of malnutrition should also be identified and given essential nutritional supplements [10].

### 3.3 Harnessing the power of technology

Technology also plays a role in the war against malnutrition in Africa, especially among urban dwellers prone to malnutrition and rural dwellers with access to mobile phones and the internet. It is an excellent means of tracking and monitoring people prone to malnutrition and even those who already suffer from it. For instance, Data Scientists and Artificial Intelligence Engineers design technological solutions to detect malnutrition on both individual and community bases. To do this, they will work with various available data on malnutrition in Africa. From these data, they will be able to build mobile apps that can predict if an individual has malnutrition and state the stage. They can also provide some basic recommendations, especially if the person does not necessarily have to see a doctor. It is established that malnutrition is a threat to Africa, and prompt actions must be taken to combat it. Harnessing the powers of technology and human interactions will go a long way in winning this war. Ensuring the collective efforts of all the involved stakeholders is also essential to achieving the goal.

### 3.4 Increase production of agricultural products

The role of agriculture in food security cannot be overlooked in a nation. If all agricultural products farmers produce can be managed extensively, Sub-Saharan Africa will be out of the burden of malnutrition. The usage of modern food production in the African agricultural system is very crucial in controlling malnutrition because; there will be no or low spoilage of agricultural products, the farmers will cultivate and produce good foods in a short time, and there will be a lot of people willing to do farming. Also, the government should contribute to increasing agricultural production by providing subsidized fertilizers to the farmers and pumping machines which can be used by the farm during the dry season. Furthermore, farmers should learn how to cultivate genetically modified foods because those foods have been modified to resist adverse weather conditions and improve yield.

### 3.5 Management and protection of the environment

Environment entities such as climate change, temperature and soil play a substantial role in controlling malnutrition. The environment controls food security via food storage, production, and transportation. Programs that will keep the farmers abreast of how to control the environment should be implemented. The government in each country in Sub-Saharan Africa should encourage the farmers to participate in these programs to protect the continuously degrading environment. Sustainable and feasible irrigation programs should be established and maintained, especially during dry seasons and in drought-affected areas.

A multisectoral approach should be used to curb malnutrition in sub-Saharan Africa. The government must work in hand with other sectors like agriculture, health, environment and education for us to have a nation without malnutrition.

## Ethical approval

Ethical approval was not required for the manuscript.

## Funding

No external funding was received.

## Authors’ contributions

AJO conceptualized the idea; AJO, ROA, ROA and EDO drafted the manuscript; AJO reviewed the manuscript; All authors approved the final manuscript for submission.

## Registration of research studies

1. Name of the registry:
2. Unique Identifying number or registration ID:
3. Hyperlink to your specific registration (must be publicly accessible and will be checked):

## Consent

Ethical approval was not required for the manuscript.

## Guarantor

The Guarantor is the one or more people who accept full responsibility for the work and/or the conduct of the study, had access to the data, and controlled the decision to publish.

## Declaration of competing interest

Authors declare no conflict of interest.
